# Cannabis and Psychosis: Genomic Methods Shed Light on Pathophysiological Processes

**DOI:** 10.1016/j.bpsgos.2026.100759

**Published:** 2026-06-11

**Authors:** Jack F.G. Underwood, Jeremy Hall

**Affiliations:** a Neuroscience and Mental Health Innovation Institute, Cardiff University, Cardiff, United Kingdom; b Department of Psychiatry, University of Oxford, Warneford Hospital, Oxford, United Kingdom

A decade ago, in a review published in *Biological Psychiatry* of epidemiological studies examining cannabis use and psychotic outcomes, the authors concluded that there was “strong enough evidence to warrant a public health message that cannabis use can increase the risk of psychotic disorders” (1). Considerable uncertainty remained over whether this process was directly causal. While cannabis use is common among adolescents and young adults, psychosis remains a rare outcome. In that same 2016 Gage *et al.* (1) review, the authors postulated that Mendelian randomization, using genetics as a fixed exposure independent of environmental confounding, could be used to probe further into the directionality of the relationship between cannabis and psychosis. This was not possible at the time because “genetic variants that robustly predict cannabis use have yet to be identified” (1), but with the benefit of advances in genetic testing, increasing sample sizes, and international collaborations, we begin to reach the threshold where such methods become practicable.

Writing in their commentary accompanying the review, Murray and Di Forti explored how different forms of exposure to cannabis may alter risk of an associated psychosis and suggested that some individuals may be more susceptible than others (2). They cited emergent reports flagging single variants potentially associated with increased risk of psychosis in individuals who smoke cannabis and posited a link between these postsynaptic genes and mechanistic proposals (2). Murray and Di Forti expressed a “…need to establish whether adolescents and people with a genetic predisposition to schizophrenia are particularly susceptible” (2) and suggested that the application of a polygenic risk score (PRS) for schizophrenia might allow for identification of correlations between that genetic risk and cannabis use.

Now, a decade later, those two authors return as part of the team behind Austin-Zimmerman *et al.*’s “Genetic pathways point to the biology underlying the association between cannabis use disorder and psychosis”, just published in *Biological Psychiatry: Global Open Science* (3). Harnessing advances in genomic methods, Austin-Zimmerman *et al.* deploy an array of approaches to explore the molecular pathways linking cannabis use disorder (CUD) and psychosis (3). These include analytical methods such as multitrait conditional and joint analysis, enabling the estimation of direct genetic effect while accounting for liability for a linked phenotype (here CUD), and MR-Clust, which uses Mendelian randomization Wald ratio estimates to create clusters of loci with shared directions of effect to both improve the effect estimate and enable annotation of variants of interest.

To undertake such analyses requires a genomic instrument with sufficient sample size to leverage small effect sizes. To construct this, Austin-Zimmerman *et al.* first meta-analyze recent genome-wide association studies (GWASs) of schizophrenia and bipolar disorder into a single psychosis phenotype (3). Such an approach is well founded because the authors cite a shared genetic correlation (*r*_g_) of 66% derived from the Mullins *et al.* 2021 GWAS of 40,000 bipolar disorder cases (4). The Grotzinger *et al.* 2025 PGC (Psychiatric Genomics Consortium) Cross-Disorder Working Group GWAS updates this with an estimated *r*_g_ of 0.678 (via linkage disequilibrium score regression) (5), suggesting that increasing sample sizes are not massively altering a relatively settled estimate. Further supporting a combined psychosis approach, the PGC Cross-Disorder work defines a 5-factor model across psychiatric disorders in which schizophrenia and bipolar disorder are tightly clustered as a single factor (5). A distinct factor contains substance use disorders, including CUD, reinforcing a rationale for examining distinct genomic risk loci and potential interaction between those 2 factors.

Recent GWASs of CUD dig deeper into this relationship using two-sample Mendelian randomization, finding evidence of a bidirectional causal relationship between CUD and schizophrenia predominantly favoring CUD to schizophrenia (6). Genomic structural equation modeling using the CUD GWAS undertaken specifically on the relationship between cannabis use, CUD, and other psychiatric disorders identified a 3-factor model in which CUD clustered with other substance use disorders, while bipolar disorder and schizophrenia loaded onto a shared psychiatric factor (7). Colocalization analyses further identified a specific genomic region near *CHRNA2* shared between CUD and schizophrenia, implicating an overlapping common biological pathway.

Previous work by Austin-Zimmerman and many of the same team partially answers a specific question postulated by Murray and Di Forti in 2016, i.e., whether cannabis interacts with the general genetic milieu underlying schizophrenia or confers risk through a more restricted set of genes associated with a specific system (2,8). In 2024, they undertook an initial study, setting out to explore whether schizophrenia genetic liability, as indexed by a PRS, in 2 samples was associated with lifetime cannabis misuse in both control individuals and individuals with psychosis and whether the schizophrenia PRS operated interactively with cannabis on risk for psychosis (8). That work confirmed that lifetime cannabis use was associated with increased odds of psychosis. That association remained when adjusting for the schizophrenia PRS, consistent with the hypothesis that cannabis use is an environmental risk factor operating at least in part independently from schizophrenia genetic risk.

Austin-Zimmerman *et al.* (3) take this a step further. Their analytic approach allows for the conditioning of the psychosis GWAS to account for genetic liability for CUD, effectively creating a structure of a psychosis GWAS in which everyone has the same standardized CUD liability. This removes the component of each single nucleotide polymorphism’s (SNP) association with psychosis that is explained by the SNP’s association with CUD, resulting in genetic loci that contribute specifically to psychosis risk. Through conditioned and unconditioned analytic runs, Austin-Zimmerman *et al.* find differential signal for loci that have impact on psychosis specifically and psychosis with CUD (3). When included in pathway analyses allowing for conditioning on CUD, 8 pathways were significantly associated with psychosis, including 2 pathways specific to the conditioned analysis, synapse organization and L-type voltage-gated calcium channel complex. They also conducted Mendelian randomization analyses that further confirmed a bidirectional causal relationship between CUD and psychosis. These approaches demonstrate and reinforce that there are both shared and separate genes contributing to CUD and psychosis risk.

Austin-Zimmerman *et al.* also apply separate pathway PGS analyses in the EU-GEI (European Network of National Schizophrenia Networks Studying Gene-Environment Interactions) dataset, which highlight a key role for glutamate and the GABAergic (gamma-aminobutyric acidergic) systems in conferring risk for psychosis in both cannabis users and cannabis nonusers (3). Cannabinoid CB_1_ receptors, on which cannabis acts, are present in both glutamatergic and GABAergic neurons, suppressing glutamate release and altering downstream tonic inhibitory control of subcortical dopamine (9). Glutamatergic dysfunction has long been hypothesized as a causal process for schizophrenia (10), and this work suggests a risk pathway involving cannabis exposure as a route to psychosis development.

It might have taken a decade since Murray and Di Forti’s commentary, but as sample sizes reach into the multidigit figures required to untangle the polygenic pleiotropy, these results help shed some light on the causative pathways underlying the relationships between cannabis and psychosis. Work such as Austin-Zimmerman *et al.*’s study is methodologically ambitious and at the forefront of current approaches. As these genomic methods mature, the findings will become even clearer, but a pattern is beginning to emerge. A person who is potentially in a critical developmental window with genetic variants that increase their susceptibility to psychosis, potentially through compromised glutamatergic signaling, who then uses cannabis heavily and further suppresses glutamate release via the CB_1_ receptor may cross a clinical threshold that they would not otherwise reach. Identification of these pathways may lead to the development of more specific drugs, but this work serves to remind us that prevention is often better than a cure. In individuals with vulnerabilities, CUD will increase risk of psychosis, and therefore, identifying individuals who may be vulnerable during key developmental periods and reducing their risk remains critical to the reduction of cannabis-associated harms.
